# Supplementary material for: High-quality superconducting α-Ta film sputtered on the heated silicon substrate
Source: Sci Rep. 2023 Aug 7;13:12810. doi: 10.1038/s41598-023-39420-y (PMC10406942; doi:10.1038/s41598-023-39420-y)
Supplement: Supplementary file 1 — Supplementary Information. [file 41598_2023_39420_MOESM1_ESM.docx]

**Supplementary Information**

Yanfu Wu^1^, Zengqian Ding^1^, Kanglin Xiong* ^1,2,a)^, Jiagui Feng*^1,2,b)^

^1^Gusu Laboratory of Materials, Suzhou, China, 215123

^2^Suzhou Institute of Nano-Tech and Nano-Bionics, CAS, Suzhou, China, 215123

^a)^ Email: klxiong2008@sinano.ac.cn

^b)^ Email: jgfeng2017@sinano.ac.cn

**Wet chemical processes of Si substrates:**

We used wet chemical cleaning method to clean the substrates before deposition. Details of the surface treatments are as follows:

1. Degreasing

Rinse in overflowing deionized water for 5 mins with agitation.

Rinse thrice in acetone bath for 5 mins with agitation.

Rinse thrice in isopropyl alcohol bath for 5 mins with agitation.

Rinse in overflowing deionized water for10 mins.

2. NH_4_OH boiling

Boil in a solution of NH_4_OH: H_2_O_2_: H_2_O (1:1:5) at 70 ^o^C for 10 mins.

(Mix NH_4_OH and H_2_O and bring to temperature. Just prior to use, add H_2_O_2_.)

Rinse in overflowing deionized water for 10 mins.

3. HCl boiling

Boil in a solution of HCl: H_2_O_2_: H_2_O (1:1:4) at 70 ^o^C for 10 mins.

(Mix HCl and H_2_O and bring to temperature. Just prior to use, add H_2_O_2_.)

Rinse in overflowing deionized water for 10 mins.

4. Piranha cleaning

Dip in a solution of H_2_SO_4_:H_2_O_2_ (2:1) for 20 mins.

Rinse in overflowing deionized water for 10 mins.

5. HF etching and HMDS passivation

Dip in a solution of HF: H_2_O (15:1) for 10 mins, then exposing the surface to an HMDS atmosphere under 120 ^o^C for 10mins immediately

**Information about the TiN_x_ buffer layer:**

Analyzing EDS elemental of Ti and N maps displayed in Fig.S1, we can see that the composition of Ti and N of α-Ta films grown at RT and 500 ^o^C are close to 1:0.34 and 1:0.28 respectively, directly illustrating that they are superconducting material. The TiN_x_ of about 100 nm thick was deposited on the Si substrate. The sputtering conditions of this sample are the same as those used to be buffer layers mentioned in the article except for deposition time. Its AFM images and temperature dependence of resistance are shown in Fig.S2. There are no obvious surface defects such as black holes in the AFM image. R became zero near 4 K. These results evidenced that the thicker TiN_x_ film is compact and superconducting. Based on the same sputtering conduction, we can reasonably speculate that the TiN_x_ buffer layer used in the article has similar compact structure and superconducting properties.

Fig.S1. EDS maps of Ti and N of α-Ta/Si films grown at RT (a, b) and 500 ^o^C (c, d).

Fig.S2. The characteristic of TiN_x_ film deposited on Si substrate. (a) Large-scale AFM image; (b) Temperature dependence of resistance.

Fig.S3. STEM of the TiN_x_/Si interfacial for Ta film grown at RT(a) and 500 ^o^C (b).
